# Supplementary material for: A single-domain antibody targets aggregation-prone region of α-synuclein to reduce synucleinopathy, rescue neurodegeneration and improve function
Source: bioRxiv. 2026 Jul 10:2026.07.07.735601. Preprint. [Version 1] doi: 10.64898/2026.07.07.735601 (PMC13370455; doi:10.64898/2026.07.07.735601)
Supplement: Supplement 1 [file NIHPP2026.07.07.735601v1-supplement-1.pdf]

bioRxiv preprint doi: https://doi.org/10.64898/2026.07.07.735601; this version posted July 10, 2026. The copyright holder for this preprint (which was not certified by peer review) is the author/funder, who has granted bioRxiv a license to display the preprint in perpetuity. It is made available under a CC-BY 4.0 International license.

Supplementary Figures

Figures S1 to S14

Other Supplementary Material

Tables S1 to S2

bioRxiv preprint doi: https://doi.org/10.64898/2026.07.07.735601; this version posted July 10, 2026. The copyright holder for this preprint (which was not certified by peer review) is the author/funder, who has granted bioRxiv a license to display the preprint in perpetuity. It is made available under a CC-BY 4.0 International license.

Supplementary figures with legends:

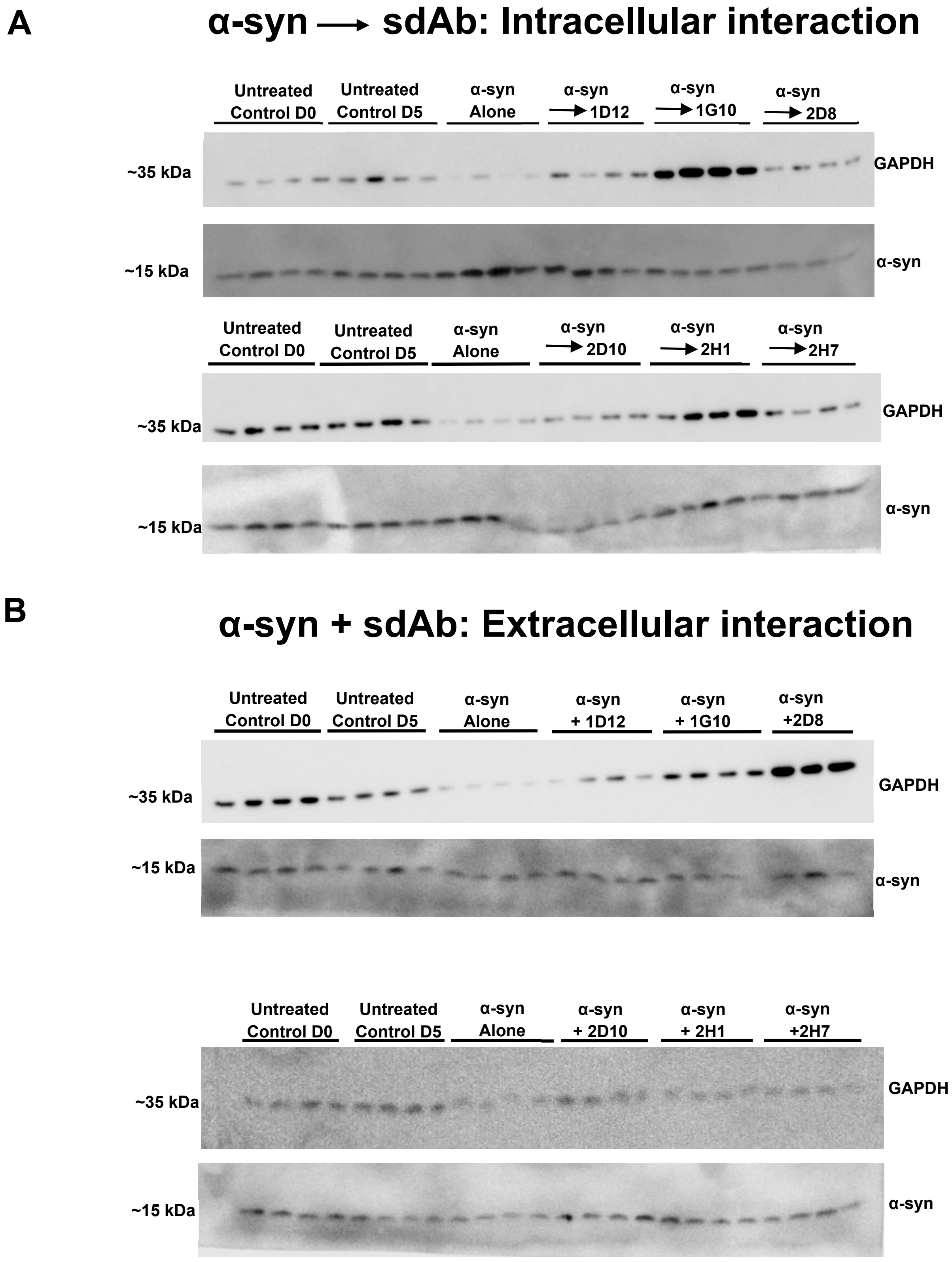

bioRxiv preprint doi: https://doi.org/10.64898/2026.07.07.735601; this version posted July 10, 2026. The copyright holder for this preprint (which was not certified by peer review) is the author/funder, who has granted bioRxiv a license to display the preprint in perpetuity. It is made available under a CC-BY 4.0 International license.

Figure S1. Representative western blots used for quantification in Fig. 1. (A-B) Immunoblots of GAPDH and α-syn levels in treated M83 cell lysate of intracellular (A) and extracellular (B) paradigms. In culture, all sdAbs except one (1D12) prevented α-syn aggregation while some prevented its toxicity.

bioRxiv preprint doi: https://doi.org/10.64898/2026.07.07.735601; this version posted July 10, 2026. The copyright holder for this preprint (which was not certified by peer review) is the author/funder, who has granted bioRxiv a license to display the preprint in perpetuity. It is made available under a CC-BY 4.0 International license.

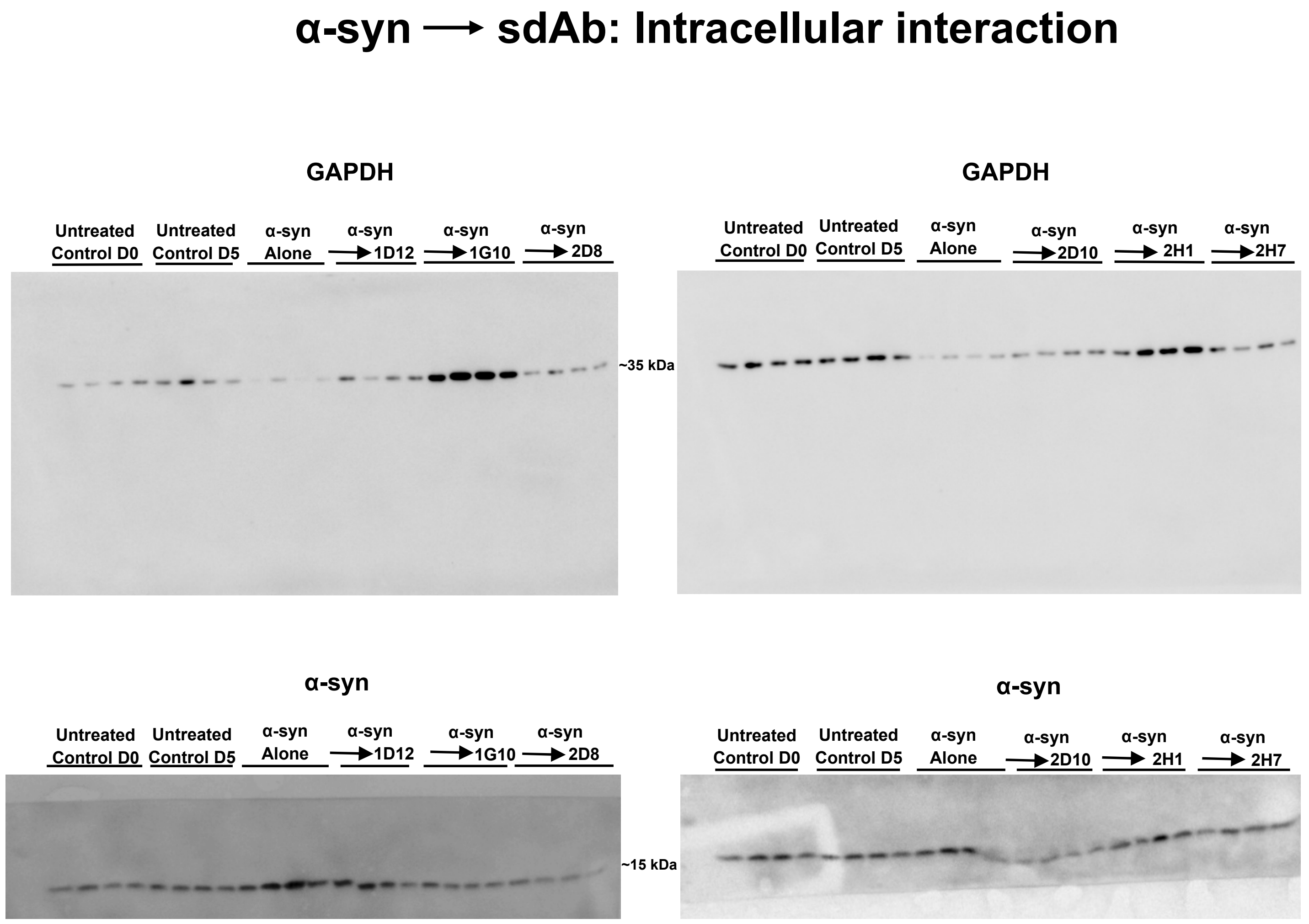

Figure S2: Complete western blots of representative blots shown in Fig. S1A. Some of the blots were cut to allow them to be reacted with different antibodies recognizing proteins at different molecular weights. Molecular weight marker was added to the first well of each gel (Precision Plus Dual Color Standards, BioRad). These bands are visible in the membrane upon transfer. The blots were developed using a chemiluminescent agent that reacts to the secondary antibody but does not always visualize the protein ladder.

bioRxiv preprint doi: https://doi.org/10.64898/2026.07.07.735601; this version posted July 10, 2026. The copyright holder for this preprint (which was not certified by peer review) is the author/funder, who has granted bioRxiv a license to display the preprint in perpetuity. It is made available under a CC-BY 4.0 International license.

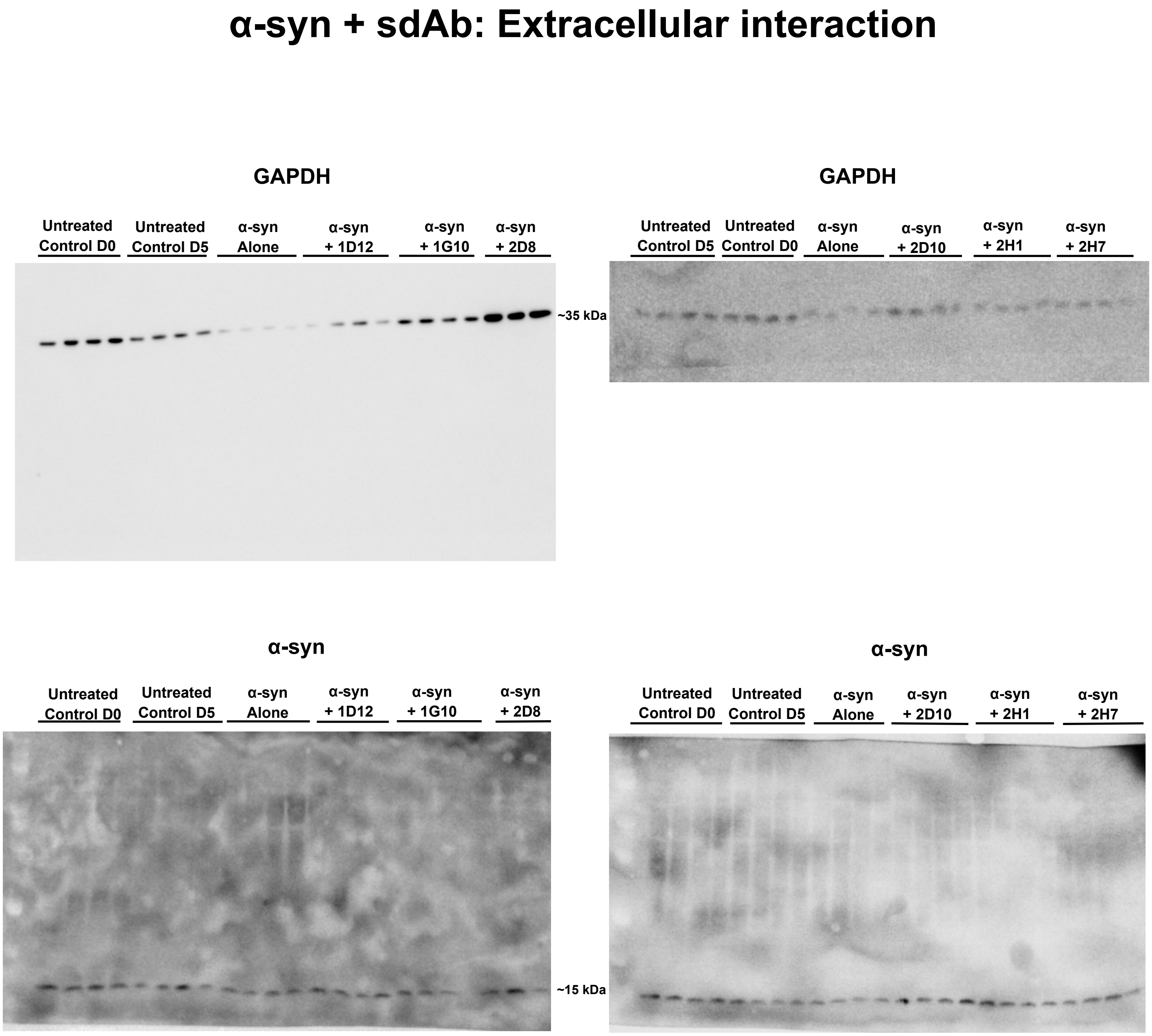

Figure S3: Complete western blots of representative blots shown in Fig. S1B. Some of the blots were cut to allow them to be reacted with different antibodies recognizing proteins at different molecular weights. Molecular weight marker was added to the first well of each gel (Precision Plus Dual Color Standards, BioRad). These bands are visible in the membrane upon transfer. The blots were developed using a chemiluminescent agent that reacts to the secondary antibody but does not always visualize the protein ladder.

bioRxiv preprint doi: https://doi.org/10.64898/2026.07.07.735601; this version posted July 10, 2026. The copyright holder for this preprint (which was not certified by peer review) is the author/funder, who has granted bioRxiv a license to display the preprint in perpetuity. It is made available under a CC-BY 4.0 International license.

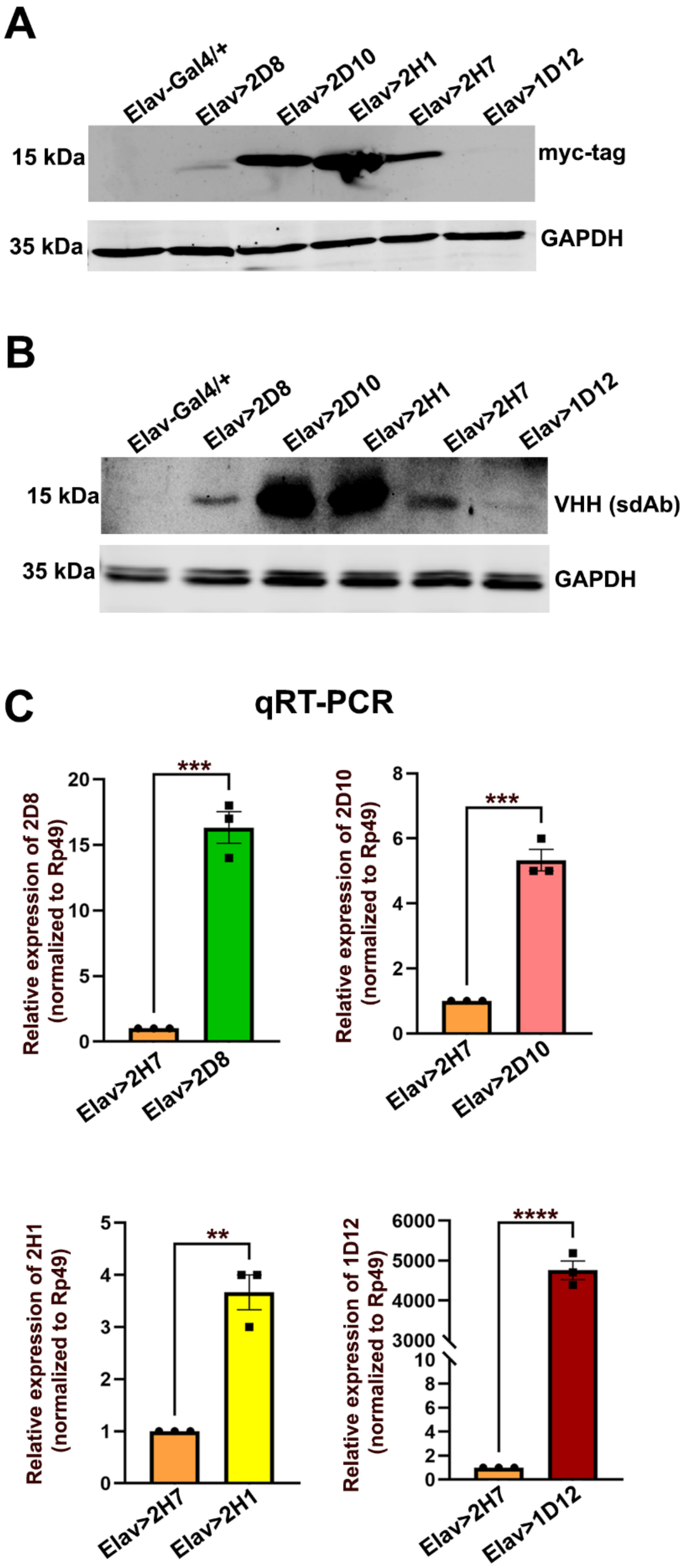

bioRxiv preprint doi: https://doi.org/10.64898/2026.07.07.735601; this version posted July 10, 2026. The copyright holder for this preprint (which was not certified by peer review) is the author/funder, who has granted bioRxiv a license to display the preprint in perpetuity. It is made available under a CC-BY 4.0 International license.

Figure S4: Pan neuronal expression of anti-α-syn sdAbs in females. (A-B) Immunoblot of protein levels of different anti-α-syn sdAbs from 5-day-old female flies detected with antibodies to myc-tag, VHH, and a GAPDH loading control. (A) Myc antibody detected robust sdAb expression in Elav>2H1, Elav>2D10, and Elav>2H7, low sdAb expression in Elav>2D8, and did not detect a signal in Elav>1D12. (B) VHH antibody detected strong expression in Elav>2D10 and Elav>2H1, followed by Elav>2H7, Elav>2D8, and Elav>1D12. (C) Quantitative RT-PCR of 5-day-old adult female fly heads driven pan-neuronally. All the sdAbs (2D8, 2D10, 2H1, 2H7, and 1D12) were clearly transcribed, with 1D12 having the highest transcript abundance. Relative expression was normalized to the housekeeping gene RP49. Bar graphs are presented as mean ± SEM. Unpaired t-test, two-tailed. $^{**}p \leq 0.01$, $^{***}p \leq 0.001$, and $^{****}p \leq 0.0001$.

bioRxiv preprint doi: https://doi.org/10.64898/2026.07.07.735601; this version posted July 10, 2026. The copyright holder for this preprint (which was not certified by peer review) is the author/funder, who has granted bioRxiv a license to display the preprint in perpetuity. It is made available under a CC-BY 4.0 International license.

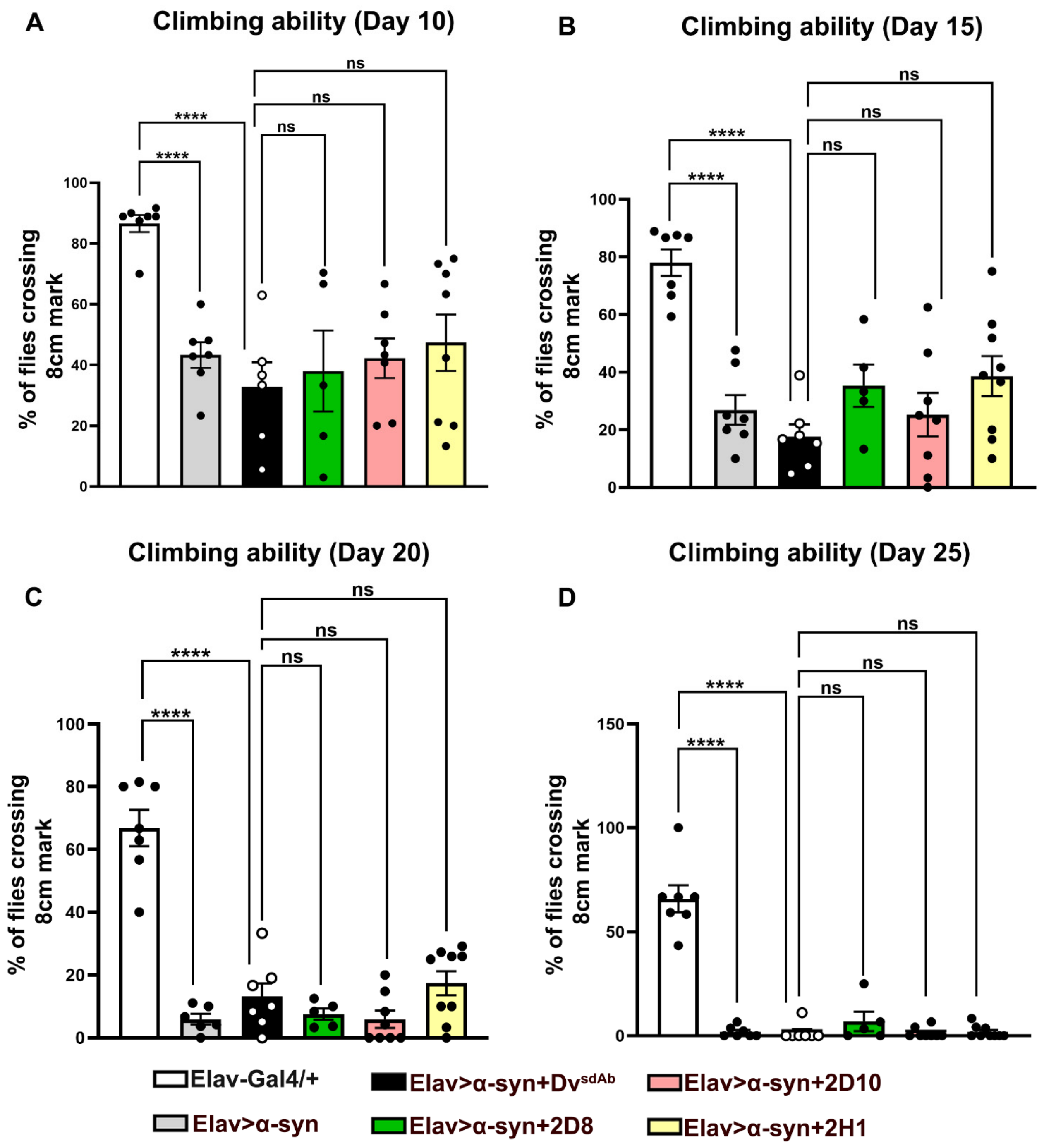

Figure S5: Pan-neuronal expression of anti-α-syn sdAbs does not rescue climbing defects in α-syn-expressing male flies. Quantitative assessment of the relative climbing ability of different age-matched adult male flies. Results from day 10, day 15, day 20, and day 25 are shown. Different genotypes are indicated by distinct colors as shown in the figure legend. Flies that do not express α-syn (white bars) climbed faster than those that do express α-syn. The grey and black bars represent flies expressing α-syn and α-syn with a control sdAb (Dv^{sdAb}). These two groups

bioRxiv preprint doi: https://doi.org/10.64898/2026.07.07.735601; this version posted July 10, 2026. The copyright holder for this preprint (which was not certified by peer review) is the author/funder, who has granted bioRxiv a license to display the preprint in perpetuity. It is made available under a CC-BY 4.0 International license.

exhibited impaired motor function at all time points, and their condition worsened with age. None of the three anti-α-syn sdAbs improved climbing ability, compared to the control Dv^{sdAb} group. Bar graphs are presented as mean ± SEM, and each data point represents an average of 5-10 flies in a vial. Genotypes and the number of flies analyzed per group: *Elav-Gal4/+* (N = 57), *Elav>α-syn* (N = 97), *Elav>α-syn+Dv*^{sdAb} (N = 63), *Elav>α-syn+2D8* (N = 42), *Elav>α-syn+2D10* (N = 69), and *Elav>α-syn+2H1* (N = 78). Two-way ANOVA, Tukey multiple-comparison test. ****$p \leq 0.0001$ and ns = non-significant.

bioRxiv preprint doi: https://doi.org/10.64898/2026.07.07.735601; this version posted July 10, 2026. The copyright holder for this preprint (which was not certified by peer review) is the author/funder, who has granted bioRxiv a license to display the preprint in perpetuity. It is made available under a CC-BY 4.0 International license.

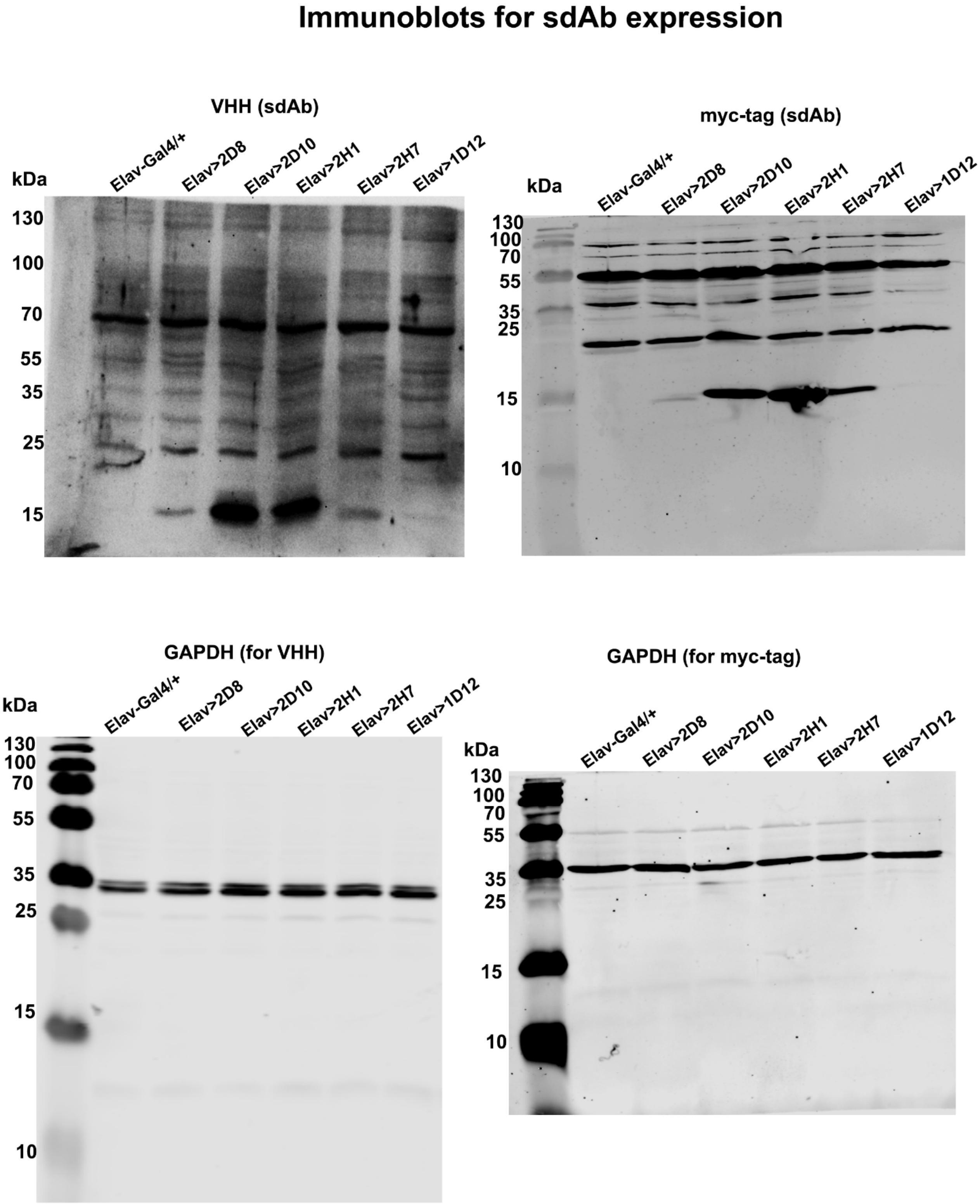

Figure S6: Uncropped western blots of representative blots shown in Fig. S4.

bioRxiv preprint doi: https://doi.org/10.64898/2026.07.07.735601; this version posted July 10, 2026. The copyright holder for this preprint (which was not certified by peer review) is the author/funder, who has granted bioRxiv a license to display the preprint in perpetuity. It is made available under a CC-BY 4.0 International license.

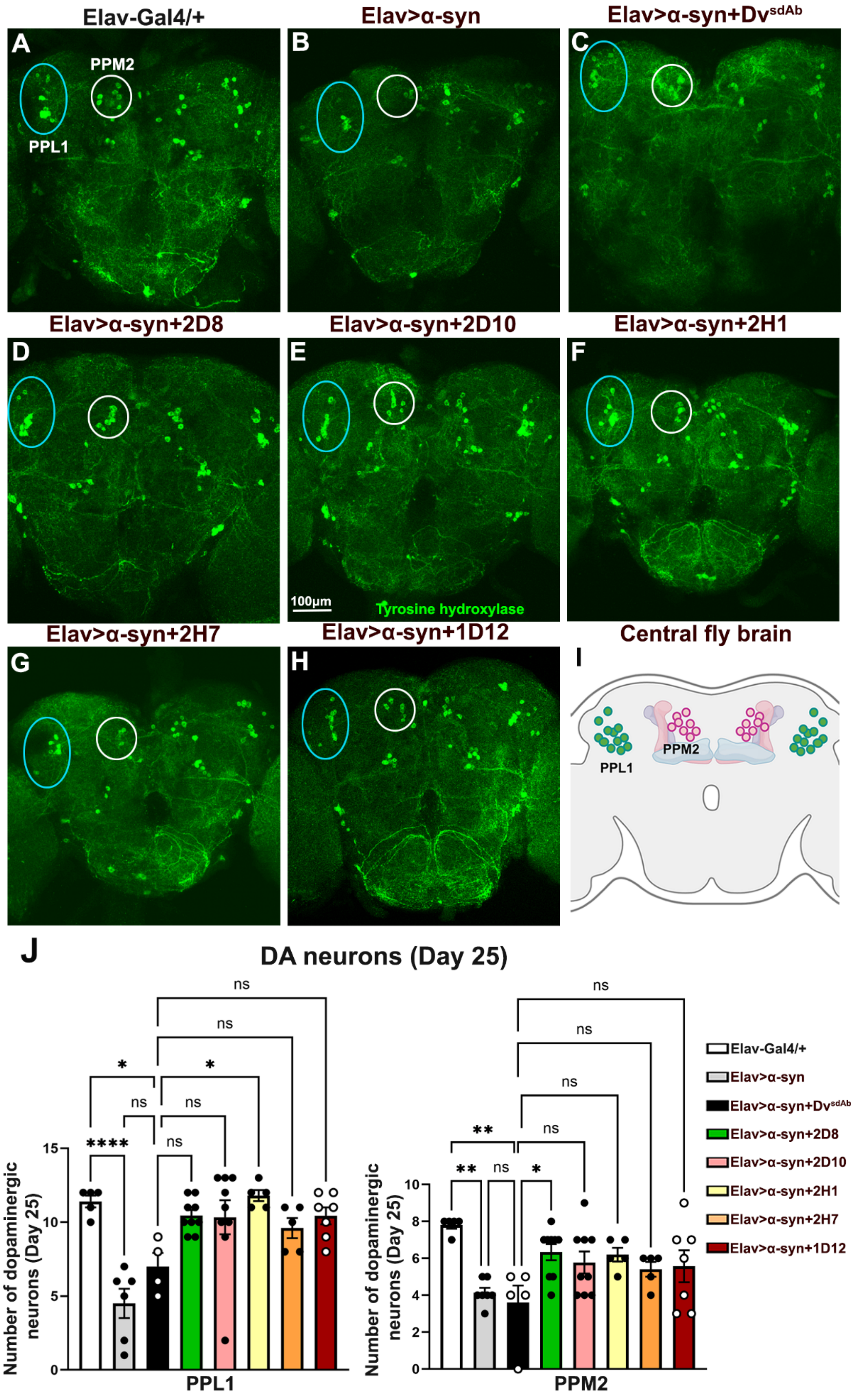

bioRxiv preprint doi: https://doi.org/10.64898/2026.07.07.735601; this version posted July 10, 2026. The copyright holder for this preprint (which was not certified by peer review) is the author/funder, who has granted bioRxiv a license to display the preprint in perpetuity. It is made available under a CC-BY 4.0 International license.

Figure S7: Pan-neuronal expression of anti-α-syn sdAbs prevents the loss of DA neurons in α-syn-expressing flies. (A-H) Fluorescence images of 25-day-old female adult brains stained with anti-tyrosine hydroxylase antibody. The marked circles outline PPL1 (blue) and PPM2 (white) neuronal clusters. (I) The schematic shows PPL1 and PPM2 neuronal clusters in an adult fly brain. (J) The average number of DA neurons in PPL1 and PPM2 neuronal clusters in different age-matched female adult brains per hemisphere (N = 5-9 per genotype). *Elav>α-syn* and *Elav>α-syn+Dv*^{sdAb} (control sdAb) flies had loss of DA neurons, and sdAb 2H1 prevented loss of PPL1, whereas sdAb 2D8 prevented the loss of PPM2 DA neurons in *Elav>α-syn* flies, whereas all other sdAbs were ineffective at 25 days post eclosion. Bar graphs are presented as mean ± SEM. One-way ANOVA, Tukey post hoc test. *$p \leq 0.05$; **$p \leq 0.01$, ****$p \leq 0.0001$ and ns = non-significant; Scale bar A-H = 100µm.

bioRxiv preprint doi: https://doi.org/10.64898/2026.07.07.735601; this version posted July 10, 2026. The copyright holder for this preprint (which was not certified by peer review) is the author/funder, who has granted bioRxiv a license to display the preprint in perpetuity. It is made available under a CC-BY 4.0 International license.

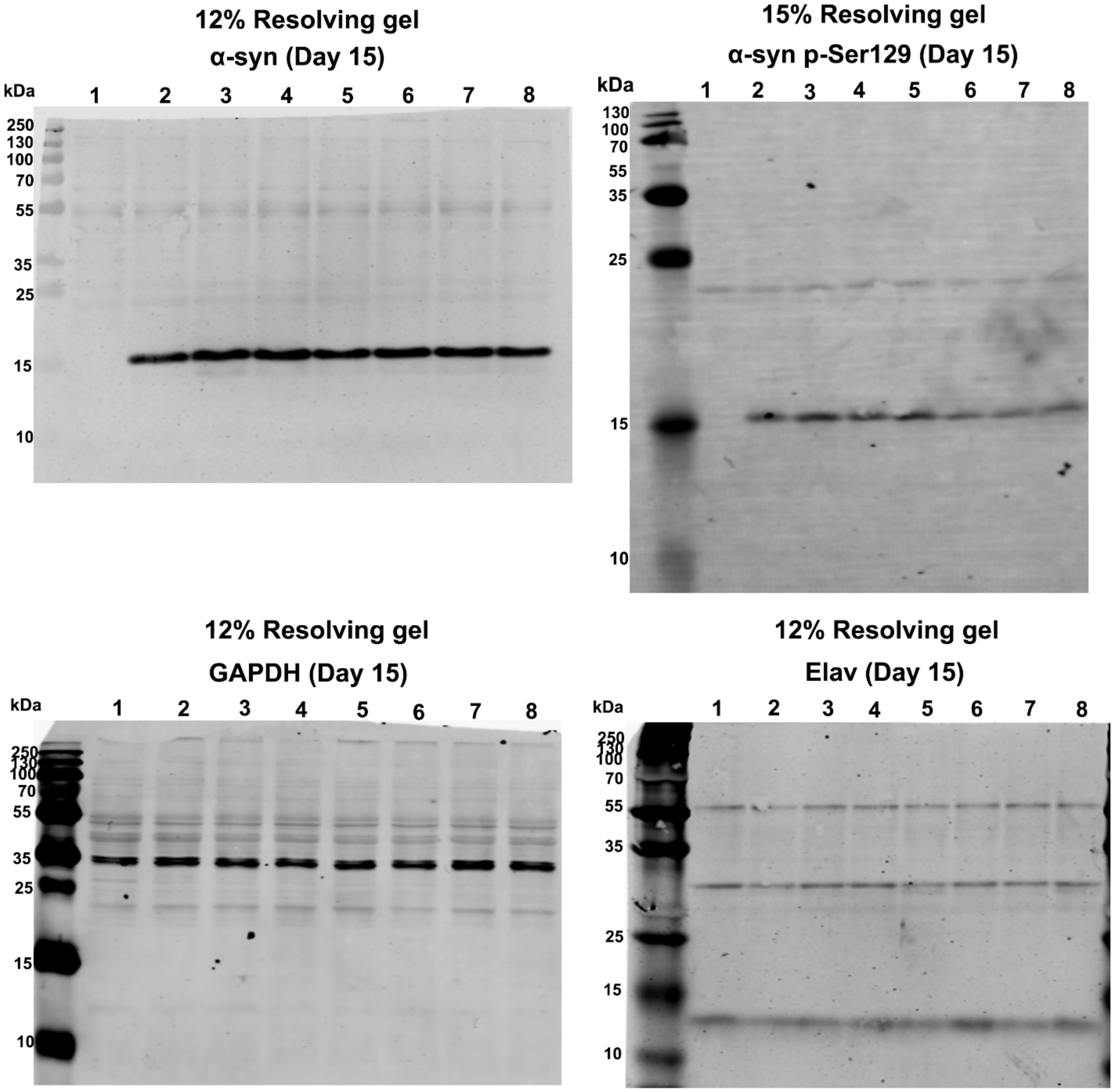

Lane 1: Elav-Gal4/+

Lane 2: Elav>α-syn

Lane 3: Elav>α-syn+Dv^{sdAb}

Lane 4: Elav>α-syn+2D8

Lane 5: Elav>α-syn+2D10

Lane 6: Elav>α-syn+2H1

Lane 7: Elav>α-syn+2H7

Lane 8: Elav>α-syn+1D12

Figure S8: Uncropped western blots of representative blots shown in Fig. 3A.

bioRxiv preprint doi: https://doi.org/10.64898/2026.07.07.735601; this version posted July 10, 2026. The copyright holder for this preprint (which was not certified by peer review) is the author/funder, who has granted bioRxiv a license to display the preprint in perpetuity. It is made available under a CC-BY 4.0 International license.

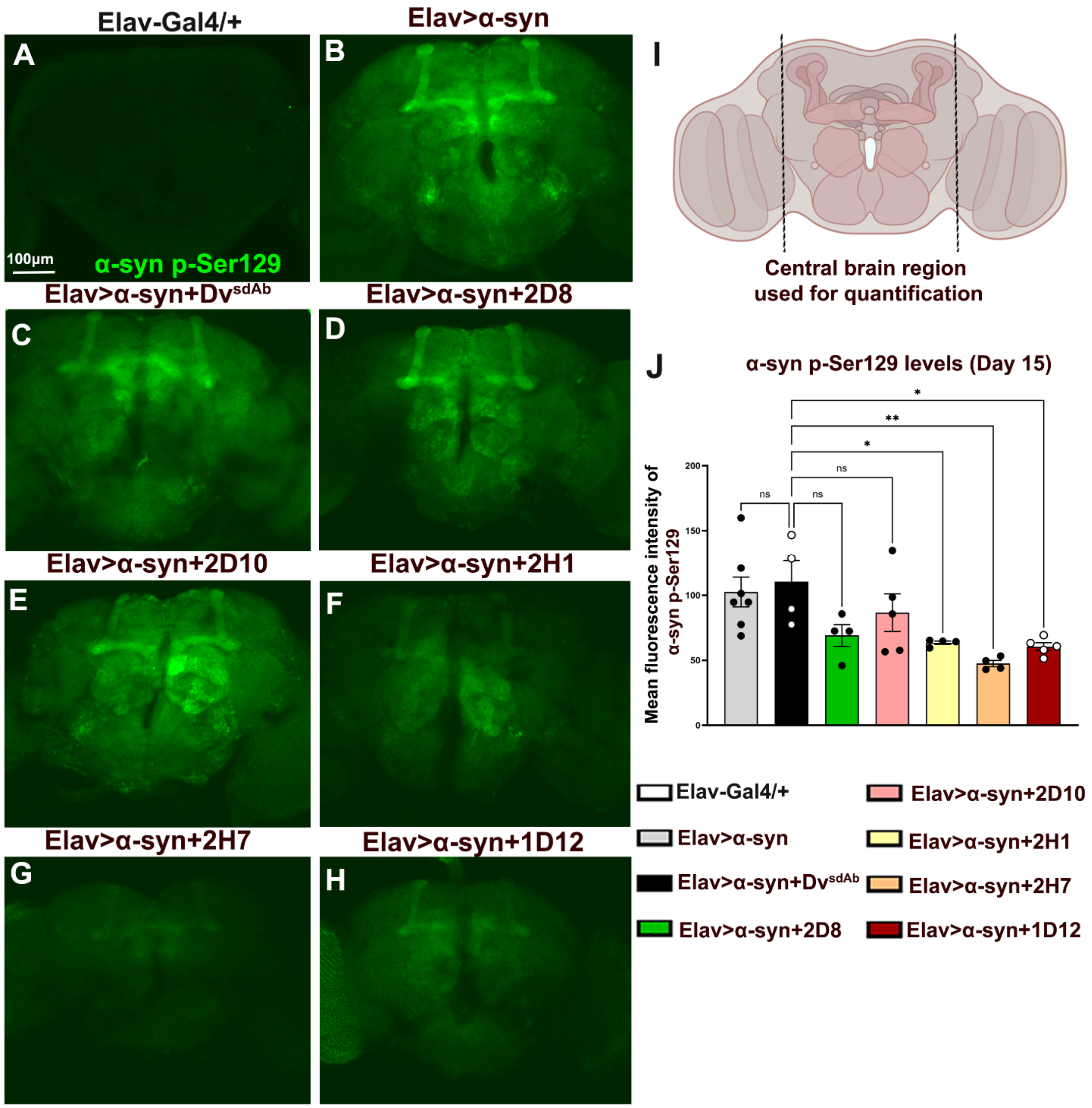

Figure S9: Neuron-specific expression of anti-α-syn sdAbs reduces pathological α-syn p-Ser129 immunoreactivity in brains of adult female flies expressing α-syn in neurons. (A-H) Confocal images of 15-day-old female adult brains stained with α-syn p-Ser129 antibody. *Elav-Gal4/+* control flies were immunonegative for α-syn p-Ser129 (A). (I) The schematic shows the central brain region of an adult fly brain. (J) Quantification of α-syn p-Ser129 immunoreactivity in the central brain region marked in I. sdAbs 2D8 and 2D10 did not reduce α-syn p-Ser129, whereas sdAbs 2H1, 2H7, and 1D12 decreased α-syn p-Ser129 levels (N = 4-7 per genotype). Scatter plot bar graphs are presented as mean ± SEM. One-way ANOVA, Dunnett's multiple comparisons test. $^{*}p \leq 0.05$, $^{**}p \leq 0.01$ and ns = non-significant; Scale bar A-H = 100µm.

bioRxiv preprint doi: https://doi.org/10.64898/2026.07.07.735601; this version posted July 10, 2026. The copyright holder for this preprint (which was not certified by peer review) is the author/funder, who has granted bioRxiv a license to display the preprint in perpetuity. It is made available under a CC-BY 4.0 International license.

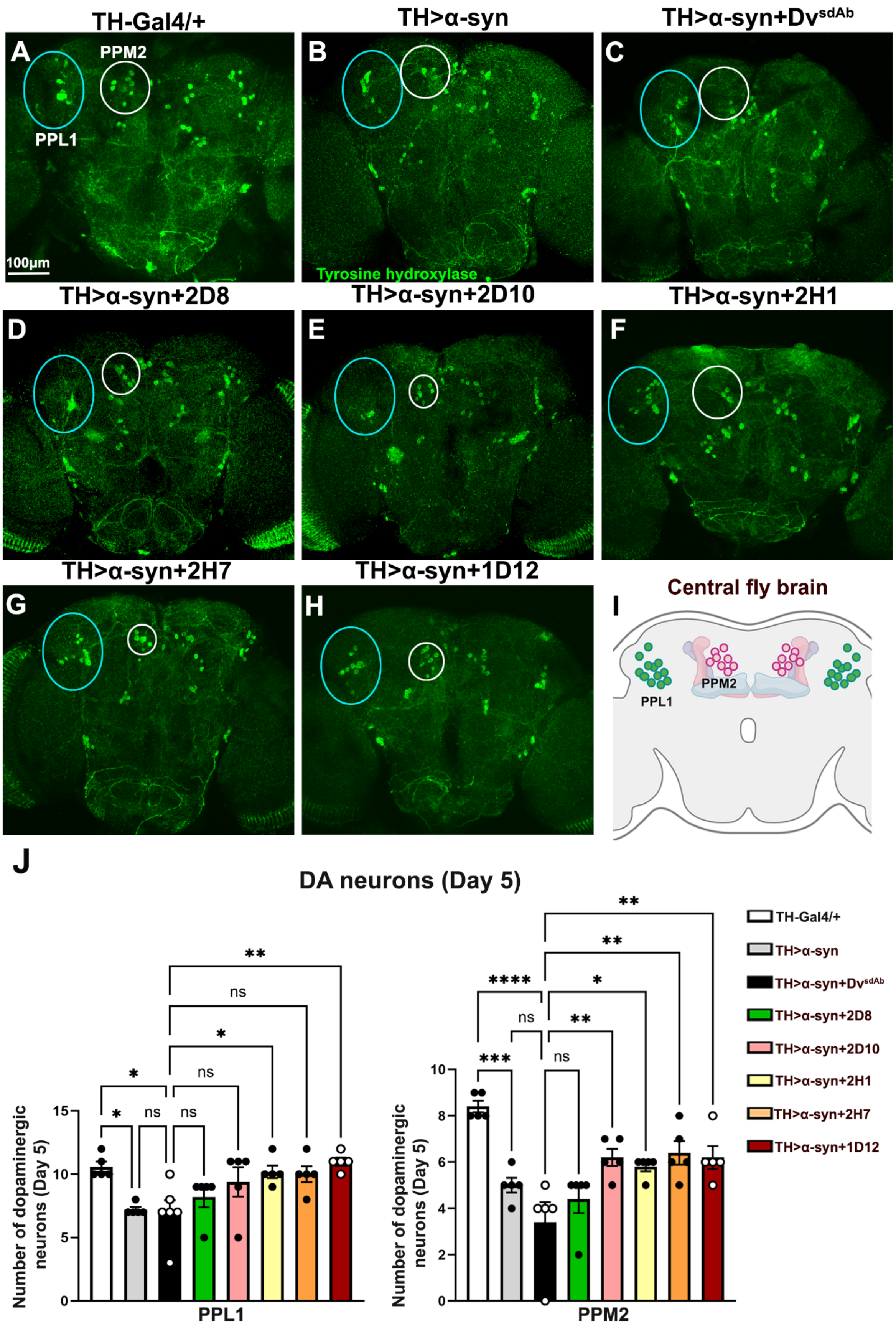

bioRxiv preprint doi: https://doi.org/10.64898/2026.07.07.735601; this version posted July 10, 2026. The copyright holder for this preprint (which was not certified by peer review) is the author/funder, who has granted bioRxiv a license to display the preprint in perpetuity. It is made available under a CC-BY 4.0 International license.

Figure S10: Targeted expression of anti-α-syn sdAbs in DA neurons expressing α-syn prevents loss of DA neurons. (A-H) Confocal images of 5-day-old female adult brains stained with anti-tyrosine hydroxylase antibody. (I) The schematic shows PPL1 and PPM2 neuronal clusters in an adult fly brain. (J) The average number of DA neurons in PPL1 and PPM2 neuronal clusters in different age-matched adult female flies per hemisphere (N = 5-6 per genotype). *TH>α-syn* and *TH>α-syn+Dv*^{sdAb} (control sdAb) flies had loss of DA neurons in different clusters. sdAbs 2H1 and 1D12 prevented loss of PPL1 DA neurons in *TH>α-syn* flies, whereas all sdAbs except 2D8 were effective in preventing PPM2 neuronal loss. Bar graphs are presented as mean ± SEM. One-way ANOVA, Tukey's multiple-comparison test. *$p \leq 0.05$, **$p \leq 0.01$, *** $p \leq 0.001$, ****$p \leq 0.0001$ and ns = non-significant; Scale bar A-H = 100µm.

bioRxiv preprint doi: https://doi.org/10.64898/2026.07.07.735601; this version posted July 10, 2026. The copyright holder for this preprint (which was not certified by peer review) is the author/funder, who has granted bioRxiv a license to display the preprint in perpetuity. It is made available under a CC-BY 4.0 International license.

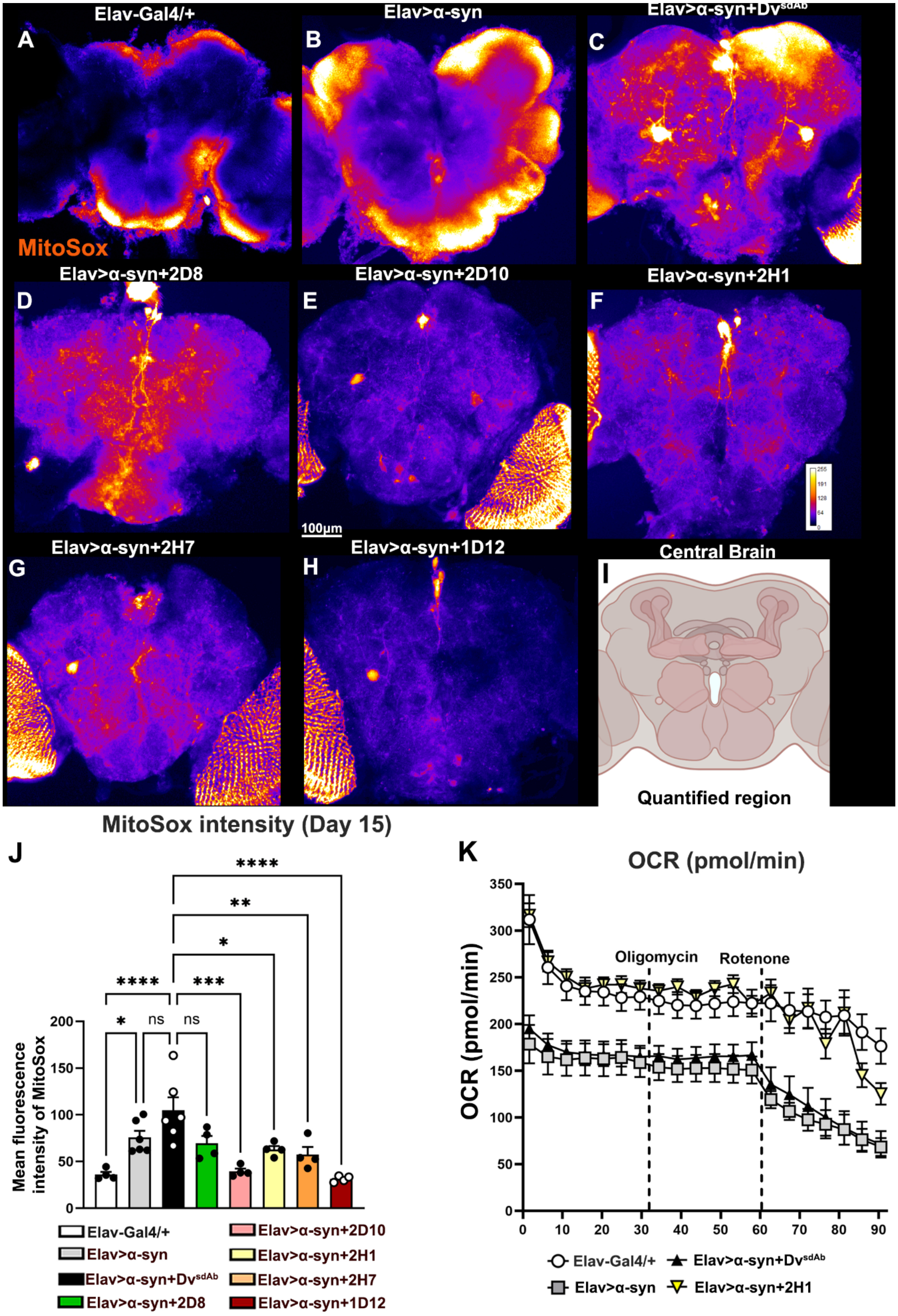

bioRxiv preprint doi: https://doi.org/10.64898/2026.07.07.735601; this version posted July 10, 2026. The copyright holder for this preprint (which was not certified by peer review) is the author/funder, who has granted bioRxiv a license to display the preprint in perpetuity. It is made available under a CC-BY 4.0 International license.

Figure S11: Anti-α-syn sdAbs reduce superoxide levels in the mitochondria and improve OCR in synucleinopathy flies. (A-H) Fluorescent images of 15-day-old female adult brains stained with MitoSox Red dye. (I) The schematic shows the central brain region of an adult fly brain. (J) The mean fluorescence intensity of MitoSOX measured in the adult brain shows that sdAb 2D8 was not effective, while sdAbs 2D10, 2H1, 2H7, and 1D12 significantly reduced superoxide levels in α-syn expressing flies (N = 4-6 per genotype). (K) Changes in the OCR levels measured after oligomycin and rotenone administration in adult brains of different genotypes (N=6-8 per genotype). 2H1 improves those levels in α-syn expressing flies to those seen in normal control flies. See Fig. 7 for a condensed version of this data with statistical analysis. Graphs show mean + or ± SEM. One-way ANOVA, Tukey's multiple comparisons test. *$p \leq 0.05$; **$p \leq 0.01$, *** $p \leq 0.001$, ****$p \leq 0.0001$ and ns = non-significant.

bioRxiv preprint doi: https://doi.org/10.64898/2026.07.07.735601; this version posted July 10, 2026. The copyright holder for this preprint (which was not certified by peer review) is the author/funder, who has granted bioRxiv a license to display the preprint in perpetuity. It is made available under a CC-BY 4.0 International license.

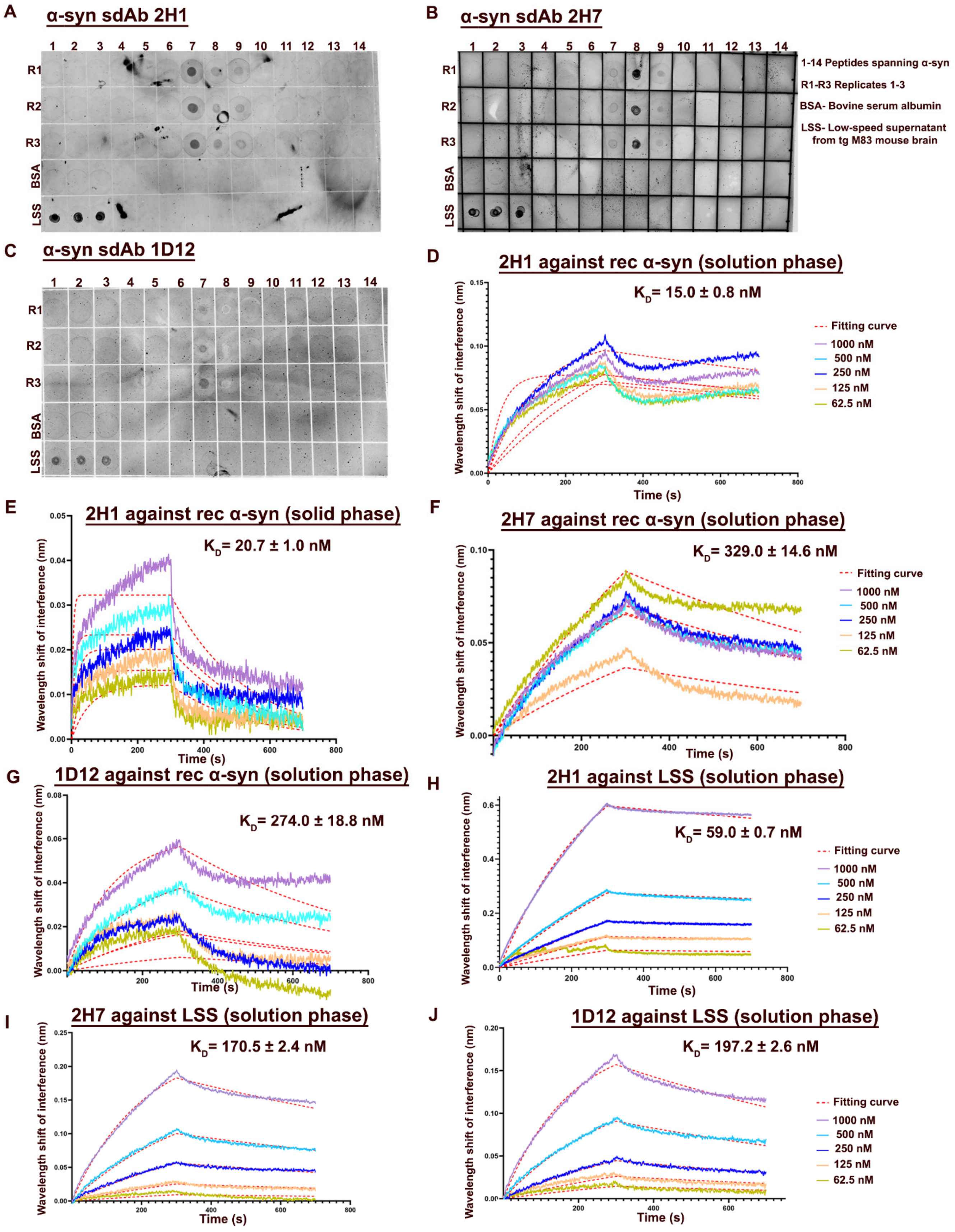

bioRxiv preprint doi: https://doi.org/10.64898/2026.07.07.735601; this version posted July 10, 2026. The copyright holder for this preprint (which was not certified by peer review) is the author/funder, who has granted bioRxiv a license to display the preprint in perpetuity. It is made available under a CC-BY 4.0 International license.

Figure S12: Epitope mapping and binding affinities of anti-α-syn sdAbs 2H1, 2H7, and 1D12 by dot blot and BLI binding affinity assay. (A-C) Dot blots of binding of sdAbs 2H1 (A), 2H7 (B), and 1D12 (C) with overlapping 14-amino-acid peptides spanning the entire α-syn protein. Negative control: bovine serum albumin (BSA). Positive control: low-speed supernatant (LSS) fractions. (D-J) Line curves of the wavelength shift of interference (in nm) for different sdAbs 2H1, 2H7, and 1D12 with recombinant α-syn (D-G) and LSS (H-J). For peptide sequences, see Table S1.

bioRxiv preprint doi: https://doi.org/10.64898/2026.07.07.735601; this version posted July 10, 2026. The copyright holder for this preprint (which was not certified by peer review) is the author/funder, who has granted bioRxiv a license to display the preprint in perpetuity. It is made available under a CC-BY 4.0 International license.

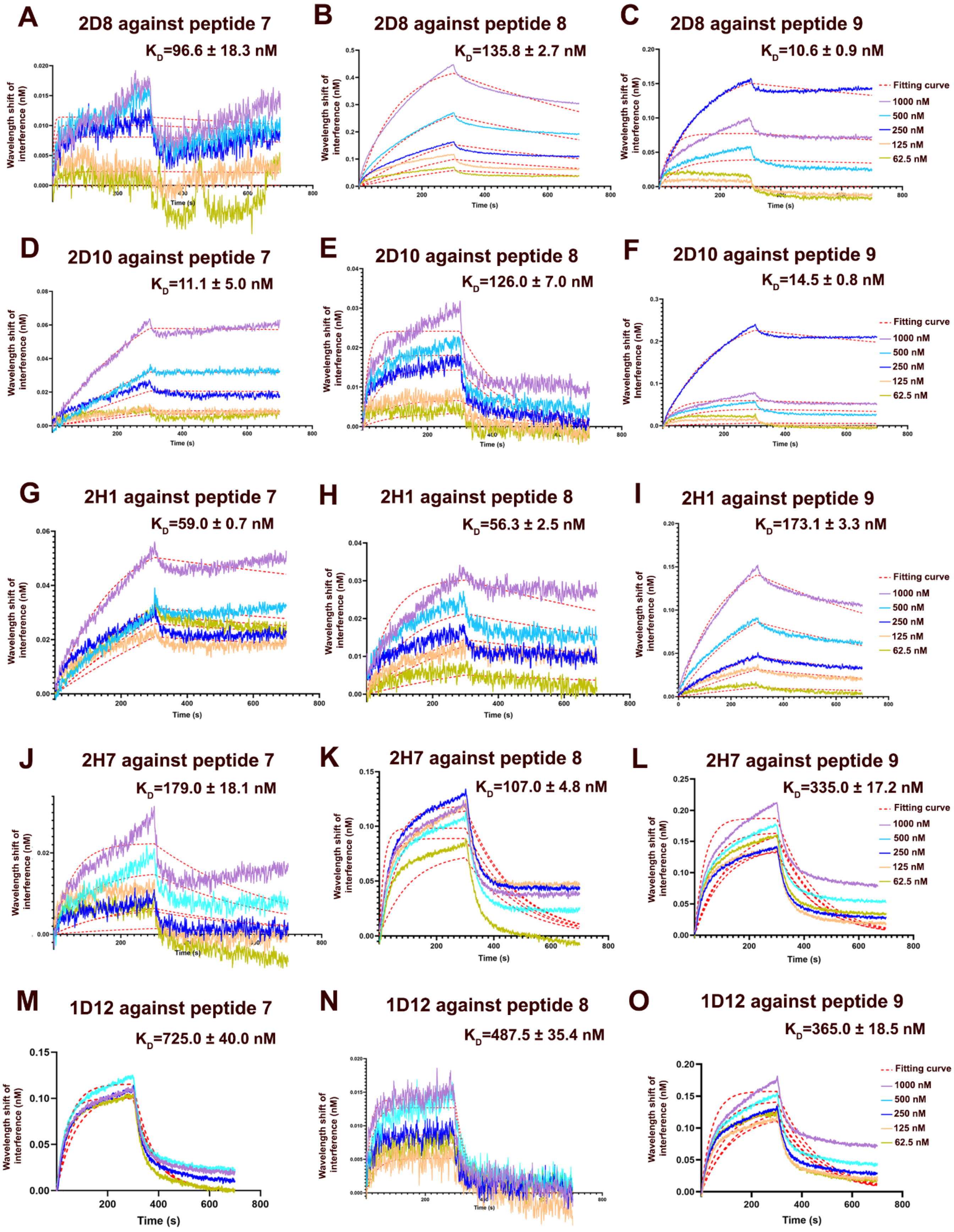

bioRxiv preprint doi: https://doi.org/10.64898/2026.07.07.735601; this version posted July 10, 2026. The copyright holder for this preprint (which was not certified by peer review) is the author/funder, who has granted bioRxiv a license to display the preprint in perpetuity. It is made available under a CC-BY 4.0 International license.

Figure S13: Binding affinities of all anti-α-syn sdAbs against peptides 7, 8, and 9 measured in solution phase by biolayer interferometry assays. (A-O) The curves illustrate the wavelength shift of interference (in nm) for different sdAbs with peptides 7, 8, and 9, indicating binding affinity. The curves illustrate the association and disassociation of sdAb with different α-syn peptides at various concentrations. The red line is the fitting curve used to determine the K_D value ± standard deviation (SD) from three independent experiments.

bioRxiv preprint doi: https://doi.org/10.64898/2026.07.07.735601; this version posted July 10, 2026. The copyright holder for this preprint (which was not certified by peer review) is the author/funder, who has granted bioRxiv a license to display the preprint in perpetuity. It is made available under a CC-BY 4.0 International license.

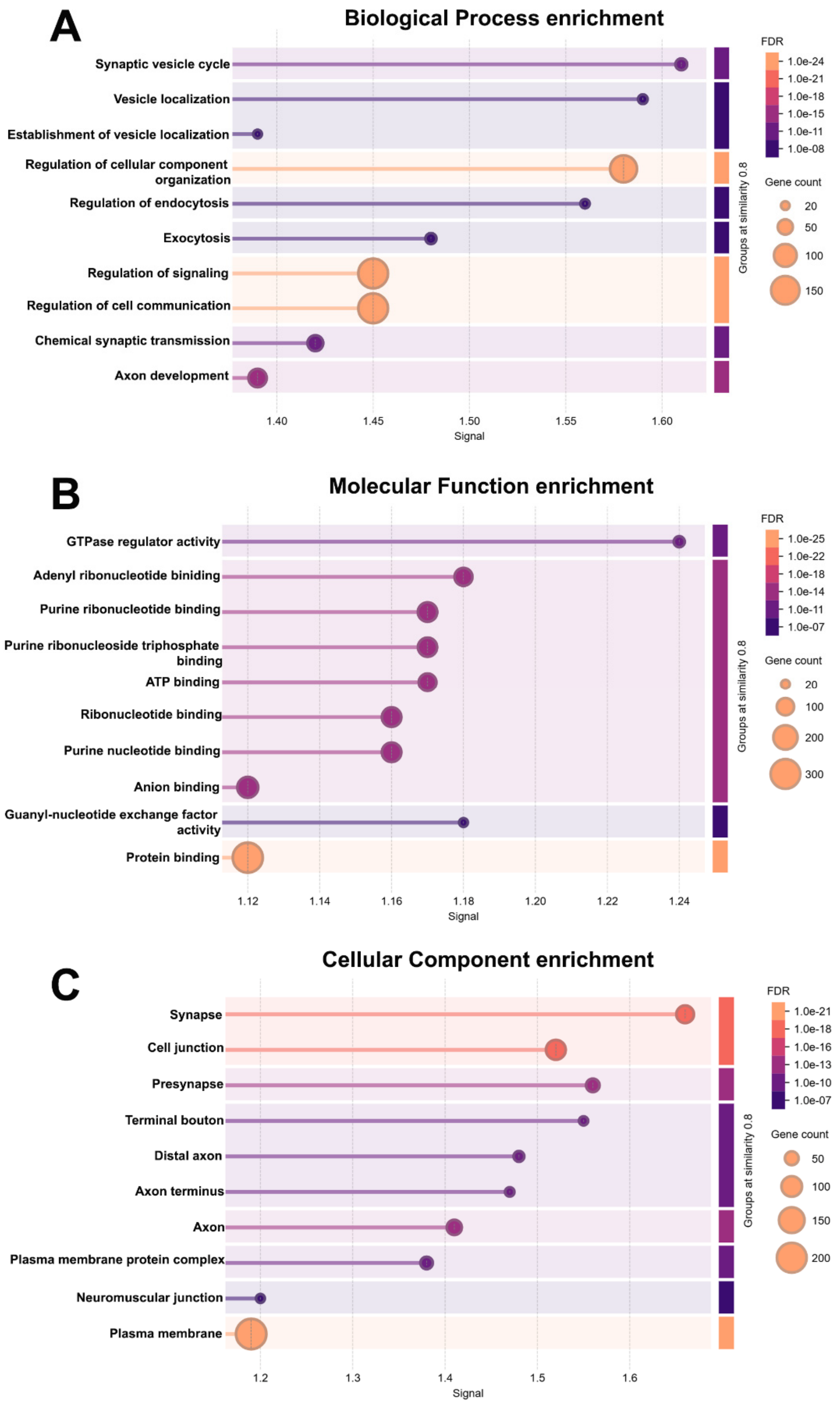

bioRxiv preprint doi: https://doi.org/10.64898/2026.07.07.735601; this version posted July 10, 2026. The copyright holder for this preprint (which was not certified by peer review) is the author/funder, who has granted bioRxiv a license to display the preprint in perpetuity. It is made available under a CC-BY 4.0 International license.

Figure S14: Biological process, molecular function, and cellular components of enriched proteins in *Elav>2H1-turboID+α-syn* flies. Pathway analysis indicates enrichment of (A) synaptic vesicle cycle, (B) GTPase regulator activity, and (C) synapse as prominent biological processes, molecular functions, and cellular components, respectively, in *Elav>2H1-turboID+α-syn* flies.

bioRxiv preprint doi: https://doi.org/10.64898/2026.07.07.735601; this version posted July 10, 2026. The copyright holder for this preprint (which was not certified by peer review) is the author/funder, who has granted bioRxiv a license to display the preprint in perpetuity. It is made available under a CC-BY 4.0 International license.

Peptide No.	Sequence
Peptide 1 (α-syn 1-15)	MDVFMKGLSKAKEGV
Peptide 2 (α-syn 11-25)	AKEGVVAAAEKTKQG
Peptide 3 (α-syn 21-35)	KTKQGVAEAAGKTKE
Peptide 4 (α-syn 31-45)	GKTKEGVLYVGSKTK
Peptide 5 (α-syn 41-55)	GSKTKEGVVHGVATV
Peptide 6 (α-syn 51-65)	GVATVAEKTKEQVTN
Peptide 7 (α-syn 61-75)	EQVTNVGGAVVTGVT
Peptide 8 (α-syn 71-85)	VTGVTAVAQKTVEGA
Peptide 9 (α-syn 81-95)	TVEGAGSIAAATGFV
Peptide 10 (α-syn 91-105)	ATGFVKKDQLGKNEE
Peptide 11 (α-syn 101-115)	GKNEEGAPQEGILED
Peptide 12 (α-syn 111-125)	GILEDMPVDPDNEAY
Peptide 13 (α-syn 121-135)	DNEAYEMPSEEGYQD
Peptide 14 (α-syn 131-140)	EGYQDYEPEA

Table S1: : Sequences of α-synuclein peptide library used for epitope mapping.
To mimic the charge state in the native protein, peptides 2-14 are acetylated on the N-terminus and peptides 1-13 are amidated on their C-terminus.
